# How Does Vertical Reading Affect Reading Speed?

**DOI:** 10.22599/bioj.149

**Published:** 2020-08-05

**Authors:** Kayleigh Porter, Gemma Arblaster

**Affiliations:** 1 Orthoptic Department, Manchester Royal Eye Hospital, GB; 2 Division of Ophthalmology and Orthoptics, Health Sciences School, University of Sheffield, GB

**Keywords:** hemianopia, vertical, reading, stroke, read

## Abstract

**Purpose:**

Vertical reading is an adaptive reading strategy sometimes used in homonymous hemianopia. This study aimed to measure horizontal and vertical reading speeds in visually normal volunteers using the Radner Reading Chart.

**Methods:**

Fifteen orthoptic students, mean age 19.7 years, took part in this repeated measures study. Participants read sentences aloud from the Radner Reading Chart horizontally and rotated vertically, to read up and down the line. Words read correctly and the time taken to read each sentence were recorded.

**Results:**

Reading speeds were calculated (words read correctly per second) for horizontal text (2.95 words per second) and for vertical text, reading up the line (1.73 words per second) and reading down the line (1.57 words per second). Reading horizontal text was significantly faster than reading vertical text. Reading horizontal text was 1.22 words per second faster than reading text vertically up (p < 0.0001) and 1.38 words per second faster than text vertically down (p < 0.0001). There was no statistically significant difference between reading text vertically up the line and vertically down the line (0.16 words per second, p = 0.42).

**Conclusion:**

Horizontal reading speed, measured with the Radner Reading Chart, was significantly faster than both vertical reading speeds. There was no significant difference between reading vertically up the line and reading vertically down the line. The slower time taken to read the vertically orientated sentences had a greater effect on reading speed than the number of errors made.

## Introduction

Stroke is estimated to occur in more than 100,000 people per year in the UK (Stroke Association, 2018). Twenty-five percent of stroke patients self-report vision problems following a stroke (Sand et al. 2015) although cohort studies of stroke survivors find the incidence of vision problems to be higher (Rowe et al. 2019). Homonymous hemianopia causes hemianopic reading deficits due to the reduced visual field, poor eye movements or perceptual difficulties (Rowe et al. 2013; Goodwin, 2014; Kerkhoff et al. 2014). To read efficiently, a person must be able to see three to four characters to the left and seven to eleven characters to the right of fixation (Kerkhoff, 2000). This is difficult for patients with visual field defects and visual strategies may be suggested to try and aid reading. Visual strategies include visual search exercises (Kerkhoff et al. 2014), visual field awareness (Rowe et al. 2013) and vertical reading (Friedman, 1982; Hepworth et al. 2019) where the print is rotated 90 degrees to be presented vertically rather than horizontally. Rotating horizontal text to read vertically is more accessible and faster to read than producing specific ‘marquee text’ where the letters are presented vertically, but upright and on top of each other (Byrne, 2002; Subramanian et al. 2014).

Studies of reading rotated text found that horizontal reading speed was 24% faster than vertical reading speed, with longer fixation durations, a greater number of small amplitude saccades, and longer gaze and regression periods during vertical reading suggested as the reasons for this difference. It was also suggested that the oculomotor system has a horizontal bias, leading to a preference to read horizontally as horizontal saccades are typically of a higher velocity than vertical saccades (Seo and Lee, 2002). Firth et al. (2007) found the rate of reading horizontal text was significantly faster than reading vertically, both when the print was rotated by 90 degrees and when the participant was rotated by 90 degrees. However only rotating right was investigated and the Wilkins Rate of Reading Test was used to measure reading speed. Saccades have been suggested as the cause of slower reading rate when reading vertically (Firth et al. 2007), which is supported by the findings that vertical saccades are less accurate than horizontal saccades (Collewijn et al. 1988a; 1988b).

Subramanian et al. (2014) measured reading speed for horizontal text and text rotated vertically to read down the line in both the central visual field and 10 degrees into the peripheral visual field of healthy volunteers. Horizontal reading speed was significantly faster than vertical reading speed in all areas of the visual field, with similar reductions in all areas of the peripheral visual field, however vertical reading speed in peripheral vision was improved with training. Training was also investigated by Calabrese et al. (2017) in patients with a central scotoma (from AMD or Stargardt’s disease) and an established preferred retinal locus to the left of their scotoma. They trained patients in horizontal (n = 5) or vertical (n = 5) reading (reading down the line) using ‘rapid serial visual presentation’ of single words for four days. Vertical and horizontal reading both improved with training. Although the improvements in vertical reading were greater than the improvements in horizontal reading, most patients still preferred horizontal reading.

de Jong et al. (2016) studied reading in patients with hemianopia or quadrantinopia, to the left or right, and age matched controls using text presented horizontally, rotated 90 degrees (reading down the line), rotated 180 degrees (reading upside down) and rotated 270 degrees (reading up the line). Reading horizontally was faster than reading rotated text. Patients with left-sided visual field defects had a greater reduction of reading speed when reading rotated text compared to right-sided visual field defects, raising the possibility that patients with right-sided visual field defects may benefit more from reading rotated text. The side of the visual defect was also found to be important in a study of stroke survivors with homonymous hemianopia (n = 7). Slower reading speeds were measured in patients with a right-sided hemianopia when text was rotated anti-clockwise (reading up the line) and in patients with a left-sided hemianopia when text was rotated clockwise (reading down the line) (Hepworth et al. 2019).

The Radner Reading Chart (Burggraaff et al. 2010) is a sentence based reading chart that has been developed as an alternative to reading charts composed of individual words presented in a random order, such as the Wilkins Rate of Reading Test. The Radner Reading Chart has been shown to provide clinically reliable and reproducible measures of reading, for both visually normal and visually impaired patients (Stifter et al. 2004; Burggraaff et al. 2010). Hepworth et al. (2019) were the first to use the Radner Reading Chart in a vertical reading study. In their feasibility study they found the Radner Reading Chart was a suitable test to measure vertical reading in stroke survivors with homonymous hemianopia, as it captured both reading speed and errors.

This study aimed to add to the existing evidence of vertical reading in a ‘normal’ population and add to the existing evidence of using the Radner Reading Chart.

## Methods

Ethical approval for the study was granted by a departmental ethics committee at the University of Sheffield. Student volunteers were recruited using an advertisement posted on a student virtual learning environment noticeboard. Volunteers with English as their first or only language, who were considered ‘visually normal’, with no amblyopia or history of previous amblyopia treatment, were recruited. The inclusion criteria were a minimum uniocular distance visual acuity of 0.1 LogMAR (ETDRS chart) wearing optical correction if required, no previous occlusion treatment and normal horizontal and vertical saccades (by observation). Participants who reported reading difficulties or specific experience of reading vertically were excluded from the study. Written informed consent was taken from all participants.

Standardised testing conditions were maintained for each participant, with all testing carried out in the same illuminated room, by the same examiner (KP), using standardised instructions. Participants maintained a straight and still head position, 40cm from the chart, by placing their chin and forehead on a rest. The Radner Reading Chart was positioned in primary position at eye level for each participant. When the chart was rotated, the chart was raised or lowered to ensure the first word of each sentence was in primary position.

**Figure 1:**
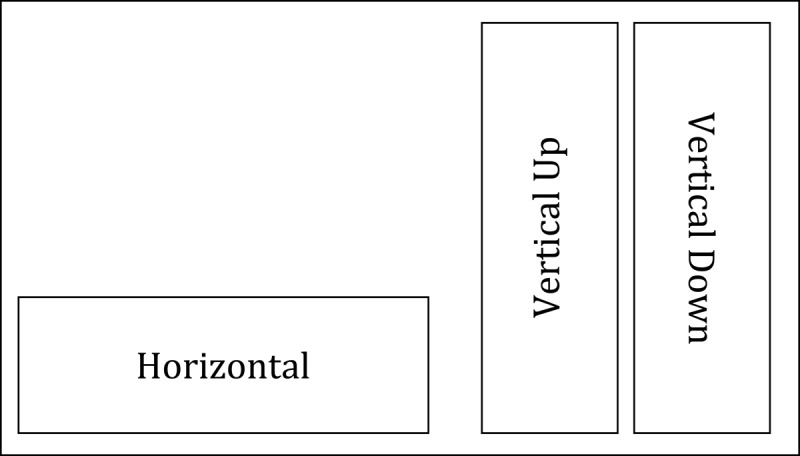
Example of text displayed at different orientations.

There are three versions of the Radner Reading Chart within the test, each with fourteen English sentences of gradually reducing size. A practice sentence was read by each participant to familiarise them with the chart format before testing. The 0.1, 0.2 and 0.3 logMAR sentences on each of the three Radner Reading Charts were used to measure reading during the experiment. The ten sentences presented were all different to avoid a learning effect during testing. Each participant read three sentences for each chart presentation (horizontal, rotated 90 degrees anticlockwise (reading up the line) and rotated 90 degrees clockwise (reading down the line) as shown in Figure 1. Testing order was randomised to minimise order effects. Participants were instructed to read all three sentences aloud as quickly as possible without making any errors. If any errors were made, they were instructed not to correct them. The time to read each sentence was recorded with a digital stopwatch and any errors made were recorded. The stopwatch was started when the chart was revealed to the participant and stopped when the participant completed the final word of the sentence. A 30 second break was given in between each chart orientation. The mean reading speed of the three sentences in each chart orientation was calculated (words read correctly per second). Parametric statistical analysis was performed using GraphPad Prism 7 as the data (words read correctly per second) was normally distributed.

## Results

Seventeen participants were screened for participation in the study. Two were excluded as they did not meet the visual acuity criteria. Fifteen participants were consented for inclusion in the study, all were female, with mean age 19.7 years, range 18–24 years.

### Reading speed

**Figure 2:**
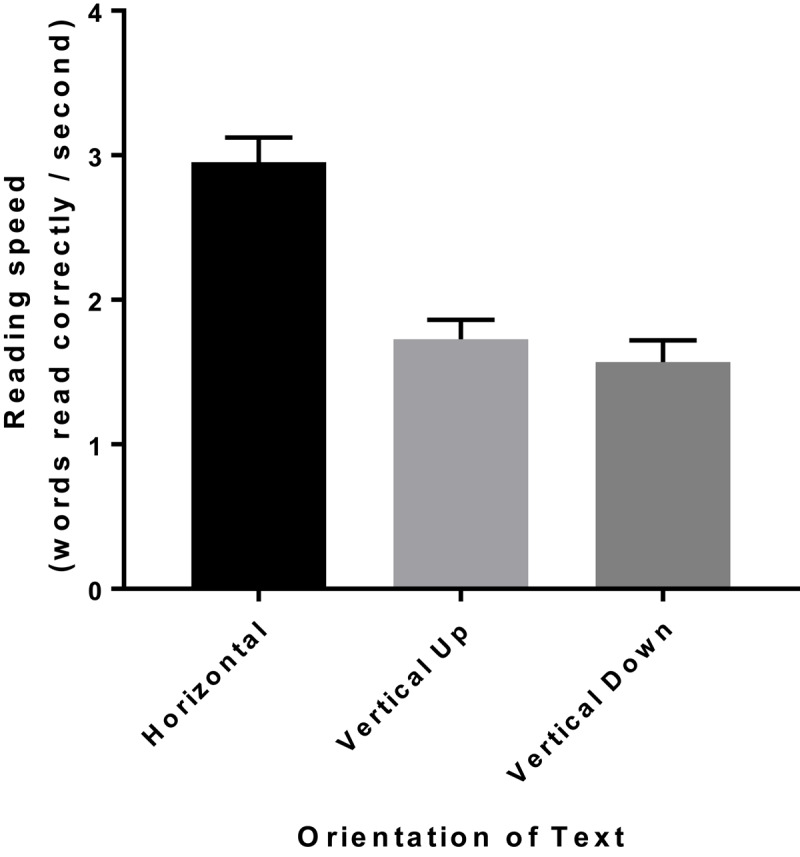
The mean and standard error reading speed (words read correctly/second) of each orientation of the Radner Reading Chart.

Mean reading speed for one sentence (words read correctly per second) for the horizontal, vertical up and vertical down orientations of the Radner Reading Chart are shown in Table 1. Data is displayed in Figure 2.

**Table 1:** Reading speed (words read correctly/second) for one sentence of the Radner Reading Chart in each of the chart orientations.

	Horizontal (Sentence Reading Speed)	Vertical Up (Sentence Reading Speed)	Vertical Down (Sentence Reading Speed)

**Mean**	2.95	1.73	1.57
**Minimum**	1.83	0.74	0.45
**Maximum**	4.07	2.63	2.92
**Standard Deviation**	0.66	0.52	0.58
**Standard Error**	0.17	0.13	0.15

The mean horizontal reading speed (2.95 words per second) was 70.5% faster than the mean vertical up reading speed (1.73 words per second) and 88% faster than the mean vertical down reading speed (1.57 words per second). Reading text horizontally meant 1.22 more words were read correctly per second compared to reading the text vertically up and 1.38 more words were read correctly per second compared to reading the text vertically down. A repeated measures ANOVA found the difference between the reading speeds of the three different orientations of the Radner Reading Chart was statistically significant (p < 0.0001). Tukey’s multiple comparison test found the difference between the reading speed of the horizontal text and the vertical up text (p < 0.0001) and the vertical down text (p < 0.0001) was statistically significant. The difference between reading the two vertical text orientations (reading up and reading down) was not statistically significant (p = 0.42).

### Reading time and accuracy

To analyse the source of the difference in the reading speed data, the mean number of words read correctly per sentence (maximum 14) and the mean time to read one sentence for each test orientation were tabulated (Table 2).

**Table 2:** Mean number of words read correctly per sentence and mean time to read one sentence for each orientation of the Radner Reading Chart.

Orientation of the Radner Reading Chart	Mean number of words read correctly per sentence (maximum 14)	Mean time to read one sentence (seconds)

Horizontal	13.87	5.11
Vertical Up	13.22	10.24
Vertical Down	12.78	10.69

A repeated measures ANOVA found the difference between the mean number of words read correctly at each orientation was statistically significant (p < 0.01). Tukey’s multiple comparison test showed the differences between the mean words read correctly for horizontal text and vertical text were statistically significant (horizontal and vertical up, p < 0.05; horizontal and vertical down, p < 0.01). The difference between the mean number of words read correctly per sentence for the vertical up and vertical down text was not statistically significant (p = 0.07).

A repeated measures ANOVA found the difference between the mean time to read one of the 14 word sentences on the Radner Reading Chart at the different orientations was statistically significant (p < 0.01). Tukey’s multiple comparison test showed the differences between the mean time to read one sentence (seconds) for horizontal and vertical text were statistically significant (horizontal and vertical up, p < 0.001; horizontal and vertical down, p < 0.001). The difference between the mean time to read one sentence (seconds) for the vertical up and vertical down text was not statistically significant (p = 0.88).

## Discussion

Healthy student volunteers had reading speeds (words read correctly/second) that were significantly faster reading horizontally (2.95 words per second) compared to reading vertically (1.73 words per second reading vertically up and 1.57 words per second reading vertically down) when tested with the Radner Reading Chart. This finding is in agreement with the results of Byrne (2002), Seo and Lee (2002), Firth et al. (2007), Yu et al. (2010) and Subramanian et al. (2014) who all investigated similar healthy volunteer populations. However, this is the first study to use the Radner Reading Chart to measure reading speed in a vertical reading study using healthy volunteers.

### Reading speed

Mean horizontal reading speed was 70.5% faster than the mean vertical reading speed, reading vertically up and 88% faster than the mean vertical reading speed, reading vertically down. In our study the difference in reading speed between reading horizontally and reading vertically was larger than in other studies, the reasons for this are unclear, but may reflect different charts and presentations, or different study designs. Seo and Lee (2002) made horizontal (left to right) and vertical (up to down) versions of Korean text and found horizontal reading speed was 24% faster than vertically reading down. Korean was traditionally presented vertically and is now typically presented horizontally, but it is unclear whether any of the participants had prior experience of reading vertically. The difference between our results and those of Seo and Lee (2002) could be due to the presentation of the text, as they projected light green text on a dark background on a screen to aid comfortable reading and reduce brightness and fatigue. Text was not rotated vertically, instead it was presented vertically. Firth et al. (2007) found horizontal reading speed to be 25% faster than text rotated 90 degrees to the right, reading down the line, using the Wilkins Rate of Reading Test. The Wilkins Rate of Reading Test presents simple words in a random order so that they do not form a sentence, yet a small number of short words are used in the test, raising the possibility that the test is easier to perform than the Radner Reading Chart used in our study. Others however, may consider the sentence structure of the Radner Reading Chart a more realistic measurement of reading and therefore easier to read than a test containing words in a random order. Yu et al. (2010) found similar increases in horizontal reading speed (81%) compared to reading vertically rotated text. However, compared to our study, they used rapid serial visual presentation of single words and flashcards of four-line blocks of text to measure reading speed.

### Direction of rotation

In our study of healthy student volunteers, the direction of rotation of the text made no significant difference to the mean reading speed, mean number of words read correctly per sentence or the mean time taken to read a sentence. Reading up and reading down the line caused a similar reduction in each measure. No statistically significant differences were found between the rotated vertically up and vertically down orientations of the Radner Reading Chart, which is similar to the findings of Byrne (2002) and Yu et al. (2010).

### Reason for reduction in vertical reading speed

The reason for the difference in reading speed between the horizontal and vertical orientations of the Radner Reading Chart in our study was mostly the additional time taken to read the vertically rotated sentences. Whilst the difference between the mean number of words read correctly per sentence was statistically significantly different when comparing reading horizontally (13.87) and reading either vertically up (13.22) or vertically down (12.78), this small difference is unlikely to be clinically significant. The difference in the mean time taken to read a horizontal sentence (5.11 seconds) compared to reading a sentence rotated and read either vertically up (10.24 seconds) or vertically down (10.69 seconds) was statistically significant. As the mean time taken to read a sentence rotated vertically was twice the mean time taken to read a horizontal sentence this would also be considered clinically significant.

Seo and Lee (2002) used a search coil to record both eye and head movements during head free reading. They recorded smaller gaze amplitudes and more frequent saccades during vertical reading and suggested these were possible reasons for slower vertical reading speed. Firth et al. (2007) theorised that saccades may be in part responsible for slower vertical reading speeds. Vertical saccades have been found to be less accurate than horizontal saccades, with greater under and over shoots, and lower peak velocity (Collewijn et al. 1988a; Collewijn et al. 1988b), supporting the view that saccade velocity and accuracy could be the reason for slower vertical reading speeds. Yu et al. (2010) instead concluded that reduced size of the visual span during vertical reading was the reason for slower vertical reading.

### Clinical relevance of findings

The results of the current study show that in healthy student volunteers with no visual problems, rotating text to read vertically significantly reduced reading speed as the time taken to read doubled. A slight increase in errors made whilst reading also occurred. The results also add to the evidence supporting the Radner Reading Chart is suitable for measuring reading speed in horizontal and vertical reading, as it allows a measurement of both reading speed and errors (Hepworth et al. 2019). This information is useful for clinicians who may be suggesting or teaching vertical reading as a strategy and may be considering using a reading test in their clinical practice. We did not specifically train our participants in vertical reading, however the findings of Subramanian et al. (2014) and Calabrese et al. (2017) suggest this may improve vertical reading speed. We agree with Hepworth et al. (2019) that future research to investigate a practice effect and the effectiveness of training as part of vertical reading would be beneficial.

### Study limitations

This study was conducted on a healthy volunteer student population; therefore, caution should be taken applying the results directly to a clinical population where vertical reading may be a useful strategy. Further studies to investigate vertical reading in patients with visual field defects would be beneficial, particularly in different sided visual field defects, as the findings of de Jong et al. (2016) and Hepworth et al. (2019) suggest vertical reading results in different sided visual field defects may differ. We aimed to evaluate the Radner Reading Chart as part of this study, but we acknowledge that other computerised methods of presenting stimuli and recording reading speed, as well as analysing the reaction times of the investigator during data collection, may have improved our measurement accuracy. We acknowledge that whilst the Radner Reading Chart contains sentences which may make it more ‘real life’ than a random word reading test, these sentences are not likely to be in context. Reading performance using the chart may therefore differ from reading contextually. We also acknowledge that our reading speed measurement did not measure comprehension of what had been read, which is an important consideration in reading.

## Conclusion

In a healthy student population rotating text to read vertically rather than horizontally increased (doubled) the time taken to read and caused a small increase in reading errors. These factors combined caused a significant reduction in reading speed (words read correctly per second) when reading vertically rotated text. The direction of rotated text (reading up or down the line) made no significant difference to reading speed. The Radner Reading Chart was suitable for measuring reading speed in horizontal and vertical reading.
